# Photocatalytic degradation of polycyclic aromatic hydrocarbons under visible light irradiation in water using TiO_2_/MgO nanocomposites

**DOI:** 10.1007/s11356-025-36055-6

**Published:** 2025-02-13

**Authors:** Iryna V. Matsukevich, Jelena Beljin, Natallia V. Kulinich, Tamara Apostolović, Snežana Maletić, Valentin Romanovski

**Affiliations:** 1 FunGlass – Center for Functional and Surface Functionalized Glass, Alexander Dubček University of Trenčin, Študentská 2, Trenčin, 911 50 Slovakia; 2 Department of Chemistry, Biochemistry and Environmental Protection, Faculty of Sciences, University of Novi Sad, Trg Dositeja Obradović 3, 21102 Novi Sad, Serbia; 3 Institute of General and Inorganic Chemistry of the National Academy of Sciences of Belarus, 9/1 Surganova str, Minsk, 220072 Belarus; 4 Department of Materials Science and Engineering, University of Virginia, Charlottesville, VA 22904 USA

**Keywords:** Magnesium titanate, Photocatalyst, Microstructure, Mesoporous powder, Polycyclic aromatic hydrocarbons

## Abstract

An extensive class of pollutants found in soil, water, and bottom sediments are categorized as polycyclic aromatic hydrocarbons. A possible method of breaking down polycyclic aromatic hydrocarbons is thought to be the photochemical approach. The potential application of mesoporous nanocomposites on TiO_2_/MgO as catalysts for the photooxidation of polycyclic aromatic hydrocarbons under the influence of visible light was assessed in this work. TiO_2_/MgO nanocomposites were successfully obtained by the self-propagating high-temperature synthesis using methotitanic acid and magnesium nitrate as metal precursors. An important step in the synthesis was the conversion of the titanium precursor into a water-soluble form with the subsequent addition of glycine and citric acid at a carbon/nitrogen (C/N) molar ratio of 0.25. This synthesis via solutions allowed the target materials with major phases of magnesium metatitanate MgTiO_3_, magnesium dititanate MgTi_2_O_5_, and magnesium titanate Mg_2_TiO_4_ to be obtained after heat treatment at 750 °C. Heterostructured mesoporous TiO_2_/MgO powders with a specific surface area of 22.0–28.4 m^2^/g had an average diameter of the predominant pores of 10–30 nm. The greatest degree of photocatalytic oxidation of fluorene, pyrene, and benzpyrene (80, 68, and 53%, respectively) was obtained when it was combined with the TiO_2_/MgTi_2_O_5_/MgTiO_3_ nanocomposite under visible light irradiation. This study showed that mesoporous TiO_2_/MgO nanocomposites could be used as photooxidation catalysts for polycyclic aromatic hydrocarbons. The maximum level of photocatalytic oxidation of polycyclic aromatic hydrocarbons in TiO_2_/MgO nanocomposites occurred at pH 7 and a photocatalyst dose of 1 mg/L under the influence of normal solar radiation.

## Introduction

Nanocomposite materials based on metal oxides are interesting because of their ability to reduce the negative impact of human economic activities on the environment and prevent air, water, and soil pollution. Thus, many modern works have shown the effectiveness of nanocomposites based on metal oxides as catalysts for the synthesis of methanol from atmospheric carbon dioxide (Huang et al. 2019; Charisio et al. 2018; Feng et al. 2018; Li et al. 2019), a sorbent of pollutants from aqueous media (Wang et al. 2022; Matsukevich et al. 2021; Liu et al. 2015; Bakhsh et al. 2022), a photocatalyst for the photodegradation of antibiotic solutions (Zhou et al. 2024) and other dissolved organic compounds (Glinskaya et al. 2023), sustainable agriculture (Periakaruppan et al. 2023; Romanovski et al. 2023) and energy systems (Romanovski et al. 2024a). Such materials can find application both for solving one of the listed problems and for a set of similar problems, for example, for restoring areas simultaneously contaminated with heavy metal ions and dyes (Ge et al. 2018).

Magnesium titanates have unique optical, electrical, and antibacterial properties, which has led to increased interest in the study of these materials. Metatitanates with the perovskite structure *M*TiO_3_ (*M* = Sr, Ba, Mg, etc.) contain oxygen vacancies and M-site vacancies due to their own nonstoichiometry (Wang et al. 2017), which increases the efficiency of photoinduced electron–hole pair separation (e^−^/h^+^) and facilitates the migration of e^−^/h^+^ pairs from the bulk to the surface (Li et al. 2023; Yang et al. 2023). Thus, MgTiO_3_ is a photocatalytic semiconductor with a band gap ranging from 2.8 to 3.7 eV (Pradhan et al. 2023; De Haart et al. 1984; Bhagwat et al. 2019; Kiani et al. 2019).

The global scientific community has focused mainly on the luminescent, optical, and electrical properties of magnesium titanate. However, work in recent years (Bhagwat et al. 2019; Kiani et al. 2019; Selvamani et al. 2021) has demonstrated the high photocatalytic activity of composites based on magnesium titanates in the process of photodegradation of organic impurities under the influence of visible light. For a magnesium titanate-based nanostructured composite with a specific surface area of 152 m^2^/g, a high sorption capacity of 241 mg/g for lead ions was achieved (Wang et al. 2015). The synergistic effects observed in heterostructured TiO_2_/MgTiO_3_ photocatalysts, including stable separation of the e-/h + pair and texturing of the powder surface, lead to a significant increase in photocatalytic activity compared to TiO_2_ with an anatase structure (Liu et al. 2018). The above findings indicate the promise of mesoporous nanocomposites based on magnesium titanate for the photodegradation of organic impurities under the influence of visible light.

Meanwhile, polycyclic aromatic hydrocarbons (PAHs) are a ubiquitous group of environmental pollutants, including air, soil, and bottom sediments (Rotondo et al. 2023; Escandar and Muñoz de la Peña 2021; Tu et al. 2022), and are included in the list of emerging pollutants (Gurgenidze and Romanovski 2023). The photochemical approach is considered a method for the decomposition of organic pollutants (Khaled et al. 2018; Zeng et al. 2023; Qu et al. 2018; Kulak and Kokorin 2023; Romanovski et al. 2024b). In addition to direct photolysis caused by the absorption of sunlight, the photodegradation of organic pollutants can occur indirectly through sensitized photolysis (Tu et al. 2022; Kulak and Kokorin 2023).

The objectives of this work were to (*i*) obtain mesoporous nanocomposites with different MgO:TiO_2_ ratios via self-propagating high-temperature synthesis (SHS) from glycine-citrate–nitrate aqueous solutions; (*ii*) study their composition, microstructure, and morphology; and (*iii*) study the efficiency of the photodegradation of PAHs (fluorene, pyrene, benzopyrene) under the influence of sunlight in the presence of synthesized TiO_2_/MgO.

## Materials and methods

### Materials and reagents

For the synthesis of TiO_2_/MgO nanocomposites, the following reagents were used: Mg(NO_3_)_2_·6H_2_O (99.9%, Aesar, Germany), H_2_TiO_3_ (99%, JSC “Vekton”, Russia), glycine (NH_2_CH_2_COOH, 99.9%, Aesar, Germany), and citric acid (C_6_H_8_O_7_, 99.9%, Aesar, Germany), which were used to obtain titanium and magnesium oxide nanocomposites by the glycine-citrate–nitrate method.

For a comparative analysis of all synthesized samples on the efficiency of photocatalytic destruction of dissolved organic substances, a solution of Direct Blue 106 dye (molecular formula C_30_H_16_Cl_2_N_4_Na_2_O_8_S_2_ and molecular mass 741.49 g/mol) was used. The photocatalytic degradation of polycyclic aromatic hydrocarbons was assessed on the best samples based on the results of a comparative analysis. Fluorene (analytical grade, Sigma-Aldrich Chemie GmbH, No. 128333), pyrene (analytical grade, Sigma-Aldrich Chemie GmbH, No. 82648), and benzopyrene (analytical grade, Sigma) were used as polycyclic aromatic hydrocarbons (Aldrich Chemie GmbH, No. B1760).

### Synthesis procedure

Composite materials in the TiO_2_/MgO system were prepared by self-propagating high-temperature synthesis from glycine-citrate–nitrate aqueous solutions using metatitanic acid H_2_TiO_3_, Mg(NO_3_)_2_·6H_2_O, citric acid C_6_H_8_O_7_, and glycine NH_2_CH_2_COOH as the starting components according to the methods described in Matsukevich et al. (2022a, 2022b, 2023). During the synthesis process, the ratio of (1–3) TiO_2_:(1–4) MgO was varied (TM1 – TiO_2_·MgO; TM2 – TiO_2_·4MgO; TM3 – 2TiO_2_·MgO; TM4 – 3TiO_2_·MgO; TM5 – 3TiO_2_·2MgO; TM6 – 3TiO_2_·4MgO). In the first stage, metatitanic acid was treated with a solution of hydrogen peroxide in an alkaline medium (ammonia solution) to convert it into a soluble form according to the following reaction:

$$\mathrm H2\mathrm{TiO}3\:+\:\mathrm{xH}2\mathrm O2\:+\:(2-\mathrm x)\mathrm{OH}-\:\rightarrow\left[\:(\mathrm{TiO}2)\mathrm x\;(\mathrm H2\mathrm O)4-\mathrm x\right]2-\mathrm x\:+\:(3/2\mathrm x\;-2)\mathrm H2\mathrm O$$

The resulting solution was mixed with a certain amount of glycine and citric acid at a carbon/nitrogen (C/N) molar ratio of 0.25. The solutions were evaporated with constant stirring on an IKA C-MAG HS-7 magnetic stirrer at a temperature of approximately 200 °C. During evaporation, the solutions thickened and turned into a gel, which was first heated in a laboratory muffle furnace at 350 °C for 5 h. The final heat treatment was carried out at a temperature of 750 °C for 5 h to obtain a white powder.

### Sample characterization

Characterization of the samples (Table 1) was carried out using X-ray phase analysis (X-ray diffraction) (X-ray diffractometer Dron-3, Cu-Kα-radiation), and the microstructure of the powders was studied using a scanning electron microscope (SEM) JEOL JSM7600F scanning electron microscope (JEOL Ltd., Japan). SEM imaging and EDS analyses were performed at an acceleration voltage of 15 kV. The crystallite sizes were estimated using the Debye–Scherrer formula for the most intense peaks of the predominant phase. The bulk density of the materials was measured in accordance with GOST 19440–94.

The adsorption properties of the samples were studied on an ASAP 2020 MP surface area and porosity analyzer (Micromeritics Instrument Corporation) from isotherms of low-temperature (− 196 °C) static physical adsorption–desorption of nitrogen. The specific surface area was determined by the single-point and multipoint Brunauer–Emmett–Teller method (A_BET_, m^2^/g). The specific pore volume (*V*_sp_, cm^3^/g), average pore diameter (*D*_sp_, nm) and pore size distribution in linear form were determined by the Barrett-Joyner-Halenda method using the desorption branch of the isotherm and the cylindrical pore model. Before analysis, the samples were evacuated for 1 h at a temperature of 100 °C and a residual pressure of 133.3·10^−3^ Pa.

### Photocatalytic activity

For a comparative analysis of all synthesized samples, a study of photocatalytic activity was carried out using the example of degradation of a solution of direct bright blue dye with a concentration of 10 mg/L and a photocatalyst dose of 100 mg/L. The change in dye concentration was monitored for 30 min with an interval of 5–10 min photocolorimetrically at a wavelength of *λ* = 590 nm with preliminary separation of the composite powder by centrifugation. The degree of photodegradation of the dye (φ_dye_, %) under the influence of UV radiation and in the presence of a catalyst was calculated using the equation:

$$\varphi_{dve}=\left(1-C_n/C_0\right)\cdot100\%,$$

where *C*_0_ is the concentration of the initial dye solution and *C*_n_ is the concentration of the dye solution after UV irradiation and separation from the catalyst. The rate constant for dye decomposition reactions (k_1_, min^−1^) was calculated in accordance with the model for pseudo-first-order reactions using the following equation:

$$\ln C_o/C=k_1\cdot\tau,$$

where *τ* is the irradiation time, min; *C*_0_ is the initial concentration, mg/L; and *C* is the concentration of the dye after time *τ*, mg/L.

The photodegradation of polycyclic aromatic hydrocarbons (fluorene, pyrene, and benzopyrene at an initial concentration of 2 mg/L) under the influence of natural sunlight was studied for three compositions with different ratios of TiO_2_ to MgO: TiO_2_·MgO, TiO_2_·4MgO and 3TiO_2_·MgO. As a control, the degradation of PAHs under sunlight without catalyst was also studied. The dose of the TiO_2_/MgO photocatalyst was varied in the range of 0.5–2.0 mg/L to determine the optimal dose. Changes in the concentration of polycyclic aromatic hydrocarbons were monitored using an Agilent 7890 gas chromatograph with an MSD 5975C mass spectrometer using an HP-5MS column (J&W Scientific) in accordance with EPA method 8270C (Beljin et al. 2023). The following chromatographic conditions were used: initial oven temperature of 55 °C for 1 min, then a heating ramp at 25 °C to 300 °C with a hold time of 3 min. The injection mode was pulsed spitless, and the inlet, quadrupole, and transfer line temperatures were 300 °C, 180 °C, and 280 °C, respectively. The PAHs were quantified in selected ion monitoring mode (SIM) using target and qualifier ions (m/z): fluorene 166, 82, and 139, pyrene 202, 174, and 101, benzo(a)pyrene 252, 126, and 113. The degree of photodegradation (destruction) of organic pollutants (φ_op_, %) under the influence of sunlight and in the presence of a catalyst was calculated using the following equation:

$${\mathrm\varphi}_{\mathrm{op}}=\left(1-{\mathrm C}_{\mathrm n}/{\mathrm C}_0\right)\cdot100\%,$$

where *C*_0_ is the initial concentration of the solution and *C*_n_ is the concentration of polycyclic aromatic hydrocarbons after 24 h, with an irradiation period with sunlight of about 16 h and a dark period without irradiation about 8 h. Notably, this experiment was carried out in the summer in a well-lit room (without additional light sources). The ambient temperature and light intensity were maintained at 29.3 ± 3.8 °C and 348 ± 97 W/m^2^, respectively.

### Photodegradation kinetics

The photodegradation kinetics were studied under the same conditions as in the previous experiment (ambient temperature of 29.3 ± 3.8 °C and daily light and dark periods of about 16 h and 8 h, respectively, with light intensity of 348 ± 97 W/m^2^). The initial concentration of PAHs was 2 mg/L. The experiments were carried out with a dose of 1 mg/L TiO_2_/MgO photocatalyst and at pH 7, as the optimal conditions determined in the photodegradation study. A series of seven samples for each of the studied materials (TM1, TM2, and TM4) as well as a series without a photocatalyst were prepared, and the contact time was varied from 0 to 72 h. The initial concentration (*C*_0_) and the remaining concentration at each contact time (*C*_t_) were determined using gas chromatography as previously described. Photodegradation kinetics were studied by monitoring the ratio of *C*_t_/*C*_0_ as a function of time to determine the time required to reach a constant concentration of PAHs and calculate the degree of photodegradation. The desorption of PAHs has been described by the following first-order kinetics:

$$\frac{S_t}{S_0}=F_{rap}\cdot e^{-k_{rap}\cdot t}+F_{slow+very\;slow}\cdot e^{-k_{slow+very\;slow}},$$

Where *S*_t_ corresponds to the amount of PAHs sorbed to the sediment (mg/kg dm) at desorption time *t* (h) and *S*_0_ is the total amount of sediment-associated PAHs immediately prior to desorption (mg/kg dm) (obtained by sample oxidation). *F*_rap_ and *F*_slow_ (*F*_slow+very slow_) (%) are the rapidly and slowly desorbing fractions, respectively, and *k*_rap_ and *k*_slow_ (*k*_slow+veryslow_) (h^−1^) are the corresponding rate constants of rapid and slow desorption, respectively. One-way ANOVA (analysis of variance) was carried out on all the results using untransformed data. *p* < 0.05 was considered to indicate statistical significance.

## Results and discussion

### TiO_2_/MgO nanocomposite characterization

**Fig. 1:**
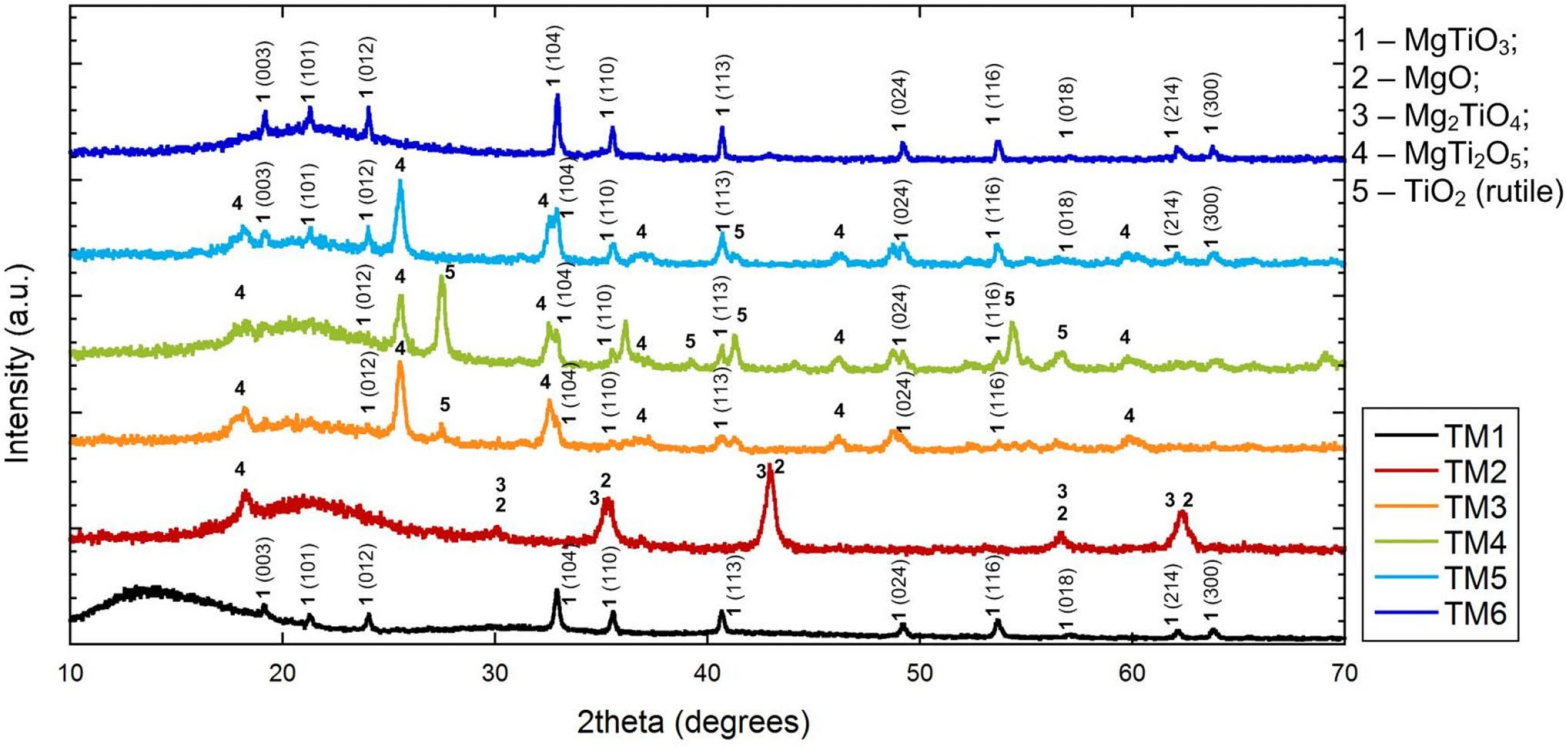
X-ray diffraction patterns of mesoporous TiO_2_/MgO composite materials after final heat treatment at 750 °C (Miller indices are indicated for the MgTiO_3_ phase)

X-ray diffraction patterns of TiO_2_/MgO composites obtained by the SHS method from aqueous solutions after final heat treatment at 750 °C demonstrate that the main phases are magnesium metatitanate (MgTiO_3_) with a perovskite structure, magnesium dititanate (MgTi_2_O_5_) with a pseudobrookite structure, which is characterized by strong distortion of cationic centers, and magnesium titanate (Mg_2_TiO_4_), which has a spinel structure. Some samples are characterized by the presence of an impurity TiO_2_ phase with a rutile structure (Fig. 1, Table 1). For samples TM2 and TM4, the crystallite sizes were not calculated since these samples contain one or more weakly crystallized phases.

**Table 1:** Characteristics of TiO2/MgO samples (the phases highlighted in the table correspond to the predominant phases for which the crystallite sizes were determined)

Sample	Composition	Phase composition	Bulk density, g/cm^3^	Crystallite sizes, nm	2theta, degrees (for the most intensive peak)
TM1	TiO_2_·MgO	**MgTiO**_**3**_	0.17	43	32.95
TM2	TiO_2_·4MgO	MgO, Mg_2_TiO_4_, TiO_2_ (impurity)	0.07	‒	
TM3	2TiO_2_·MgO	MgTiO_3_, **MgTi**_**2**_**O**_**5**_, TiO_2_	0.19	31	25.5
TM4	3TiO_2_·MgO	MgTi_2_O_5_, MgTiO_3_, TiO_2_	0.57	‒	
TM5	3TiO_2_·2MgO	MgTi_2_O_5_, **MgTiO**_**3**_, TiO_2_	0.16	55	25.5
TM6	3TiO_2_·4MgO	**MgTiO**_**3**_, Mg_2_TiO_4_	0.13	46	35.5

After identifying the most promising samples from the six synthesized TM1-6 materials, an experiment was conducted to determine the efficiency of destruction of the direct bright blue dye. A study of the photocatalytic oxidation of direct bright blue light in the presence of TiO_2_/MgO composites showed that the obtained samples indeed had a fairly high photocatalytic activity (Table 2), and the obtained values were very close to each other. For this reason, samples with three different ratios of TiO_2_ to MgO were selected for further studies: TiO_2_·MgO, TiO_2_·4MgO, and 3TiO_2_·MgO.

**Table 2:** The degree of photodegradation of direct bright blue dye under the influence of UV radiation and the rate constant of decomposition reactions after 30 min

Sample	φ_dye_, %	*k*_1_, min^−1^
TM1	69.5	0.0396
TM2	65.2	0.0352
TM3	69.1	0.0392
TM4	73.1	0.0437
TM5	70.9	0.0412
TM6	70.1	0.0403

**Fig. 2:**
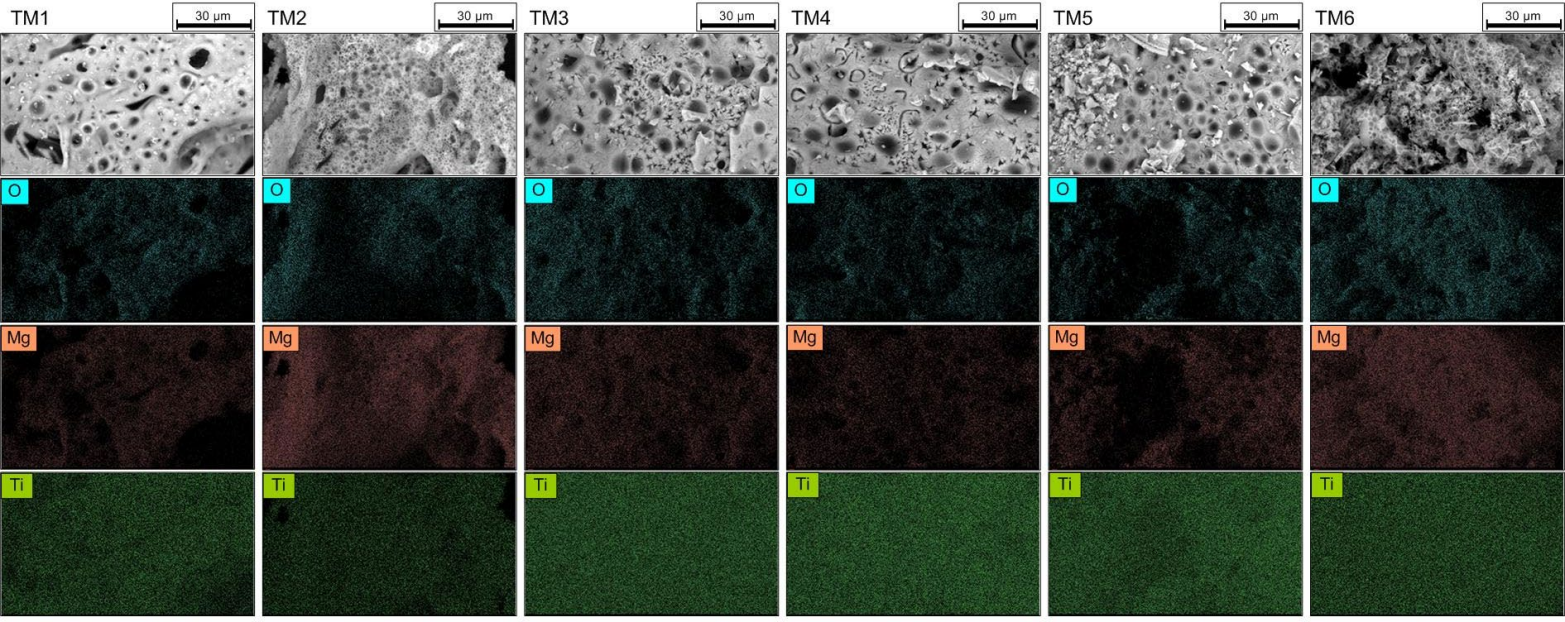
SEM images of the obtained magnesium titanate samples

Magnesium titanates have a cellular microstructure with a developed system of open and closed pores (Fig. 2), which is formed as a result of the release of a large volume of gaseous products during the SHS process. The values of bulk density increase noticeably with increasing TiO_2_ content in the composition of the heterooxide systems, while the minimum value was characteristic of the TiO_2_·4MgO sample and was 0.07 g/cm^3^ (Table 1).

**Fig. 3:**
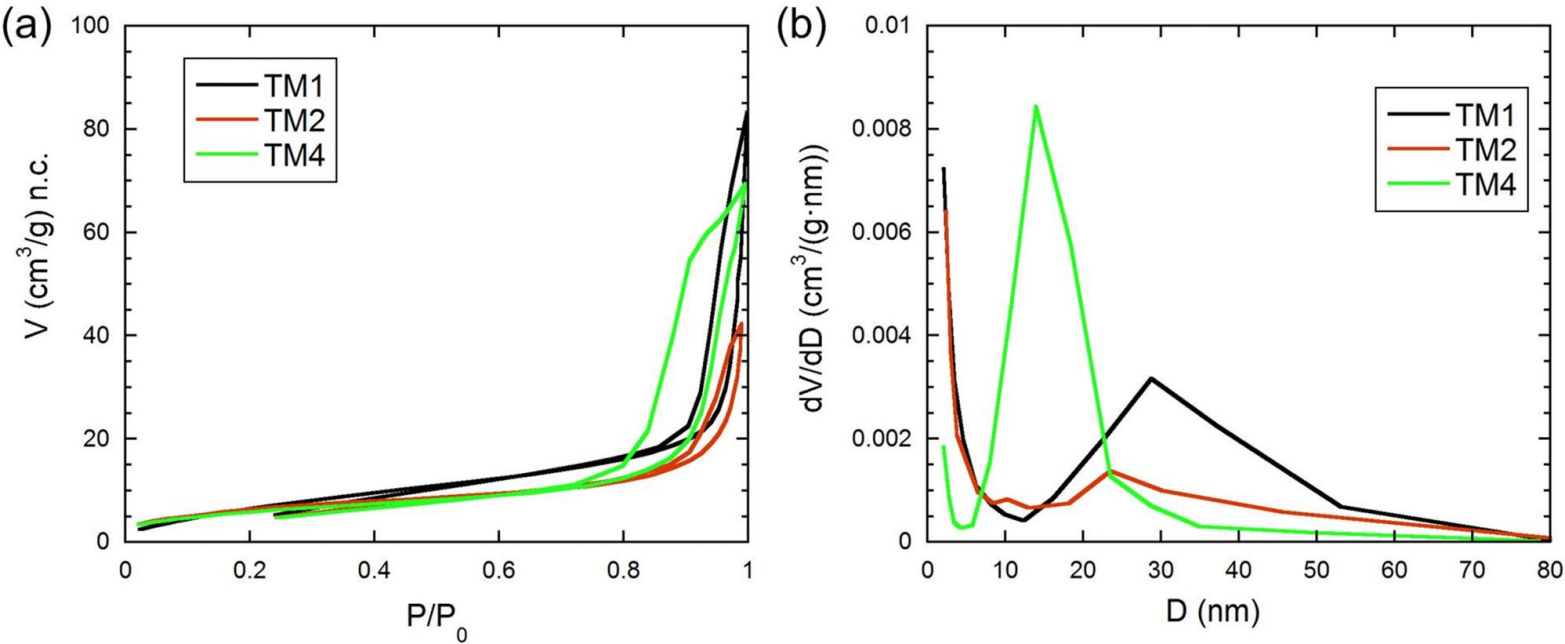
Low-temperature nitrogen adsorption–desorption isotherms (**a**) and differential mesopore size distributions (**b**) of nanocomposites based on magnesium titanate

The nitrogen adsorption–desorption isotherms of the nanocomposites based on magnesium titanate are type IVa isotherms according to the IUPAC classification (Thommes et al. 2015), which are characteristic of mesoporous adsorbents with a pore size of 2 ≤ D ≤ 50 nm (Fig. 3). Low-temperature nitrogen adsorption–desorption isotherms exhibit pronounced capillary-condensation hysteresis loops caused by nonrigid aggregates of lamellar particles and do not plateau at high values of relative pressure P/P_0_. According to the shape of the capillary-condensation hysteresis loops on the isotherms in the region of polymolecular adsorption, the samples contain pores equivalent to cylindrical and slit-like mesopores.

The area of the hysteresis loops increases with increasing TiO_2_ content, which indicates an increase in porosity in this series (Fig. 3). On this basis, it can be assumed that the addition of TiO_2_ to MgO facilitates pore formation during the synthesis process. The values of the specific surface area and average pore diameter of the studied samples depend on the composition and vary in the ranges of 22.0–28.4 m^2^/g and 17–24 nm, respectively (Table 3). The obtained specific surface areas are almost 2 times greater than those of similar MgTiO_3_/MgTi_2_O_5_/TiO_2_ materials obtained by the authors (Meng et al. 2017).

**Table 3:** Specific surface area (ABET), pore volume (Vsp), and average pore diameter (Dsp) of nanocomposites based on magnesium titanate

Sample	Gross formula	*A*_BET_, m^2^/g	*V*_sp_, cm^3^/g	*D*_sp_, nm
TM1	TiO_2_·MgO	28.4	0.13	20
TM2	TiO_2_·4MgO	24.2	0.06	17
TM4	3TiO_2_·MgO	22.0	0.11	24

The mesopore size distribution curves demonstrate the homogeneity of the mesopores in the studied samples, with predominant diameters of 10–30 nm. The most uniformly mesoporous of those studied is sample TM4 with the composition 3TiO_2_·MgO, which has the largest average pore diameter of 24 nm (Table 3).

### Photocatalytic degradation of polycyclic aromatic hydrocarbons in water media

**Fig. 4:**
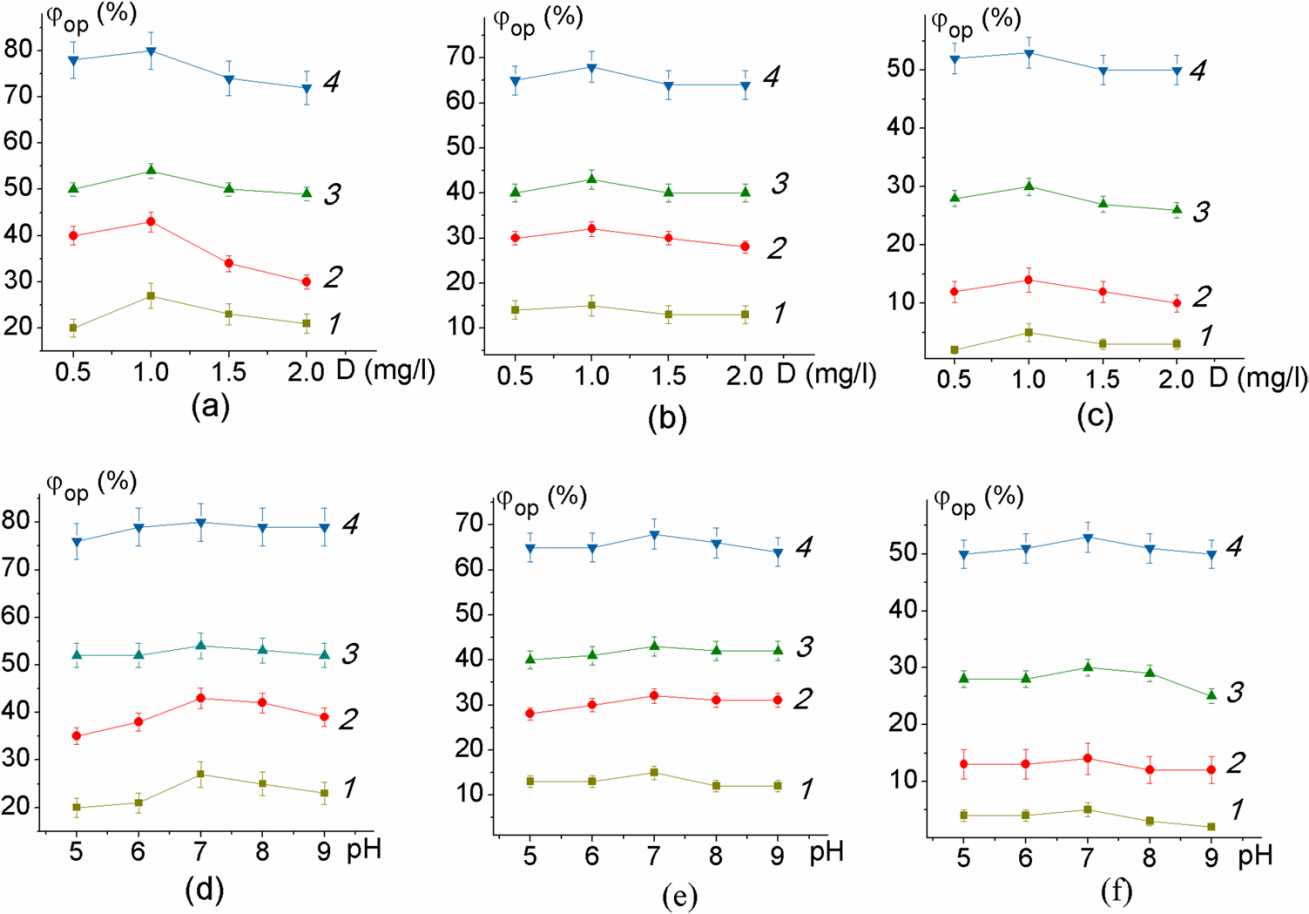
Degree of photodegradation of fluorene (**a**, **d**), pyrene (**b**, **e**), and benzo(a)pyrene (**c**, **f**) under the influence of sunlight depending on the dose of photocatalyst (**a**–**c**) and pH (**d**, **e**). 1 — without photocatalyst, 2 — sample TM1, 3 — sample TM2, 4 — sample TM4

A study of the process of photocatalytic oxidation of PAHs in the presence of TiO_2_/MgO composites showed that the resulting samples had fairly high photocatalytic activity (Fig. 4).

Using the dependence of the degree of photocatalytic oxidation of PAHs in the presence of TiO_2_/MgO nanocomposites (Fig. 4), it was established that the highest efficiency of decomposition is achieved with a photocatalyst dose of 1 mg/L and at pH 7. After 24 h of irradiation with sunlight under these conditions, the degree of photodegradation in the presence of sample TM4 containing MgTiO_3_, MgTi_2_O_5_, and TiO_2_ showed the highest values — 80, 68, and 53% for fluorene, pyrene, and benzopyrene, respectively. The significant superiority of the heterostructure sample TM4 can be explained as follows: due to suitable matching of the edges of the conduction bands in the system of semiconductor oxides TiO_2_/MgTi_2_O_5_/MgTiO_3_ (Wang et al., 2016; Yang et al. 2018), stable separation of the e^−^/h^+^ pair is achieved, which results in transfer to the crystallite surface charges necessary for the formation of OH-radicals. In addition, compared to others, sample TM4 contains a large amount of titanium dioxide with a rutile structure, which demonstrates high activity in the processes of photocatalytic oxidation of organic impurities in aqueous solutions (Gupta and Tripathi 2011).

Previous research papers have indicated the significant role of TiO_2_ in the photodegradation removal of PAHs (Pawar et al. 2023; Hyunwoong et al. 2013). McQueen et al. (2021) reported that pyrene significantly degraded by 91% over 360 min with TiO_2_ under UV/light irradiation. According to, Zheng et al. (2014) anthracene caused 99% degradation in 120 min with TiO_2_ under visible light. Phenanthrene degraded by 75% within 15 min using TiO_2_ under UV light, as reported by Zhang et al. (2011).

Fluoranthene showed 88% degradation in 120 min with unspecified phase TiO_2_ under UV/solar irradiation, as noted by Aziz et al. (2021). Benzo[α]pyrene demonstrated 85% degradation in 120 min using anatase TiO_2_ under UV/light irradiation, as reported by Bai et al. (2017). Methyl phenanthrene exhibited a lower degradation of 40% over 60 min with anatase TiO_2_ under UV/light, according to Soni et al. (2017). Luo et al. (2015a, b) reported that benzo(a)anthracene achieved a remarkable 99.7% degradation in 15.3 min using anatase TiO_2_ under UV light. Chrysene had the lowest degradation of 19% in 20 min with anatase TiO_2_ under UV/light, as reported by Shaban (Shaban 2019). Finally, benzo[g,h,i]perylene showed a high degradation of 99% over 1440 min with unspecified phase TiO_2_ under UV/light, according to Sohara et al. (2021).

Due to the active photocatalyst’s dilution with magnesium oxide, the nanocomposite TiO_2_/MgTi_2_O_5_/MgTiO_3_ exhibited the highest degree of photo-catalytic oxidation of fluorene, at 80%. We can presume that our findings generally agree with the data from the literature.

### Photodegradation kinetics

**Fig. 5:**
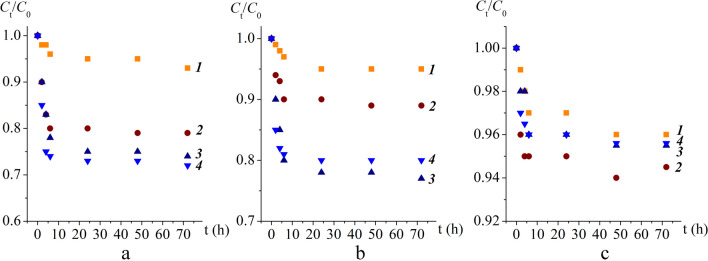
Degradation kinetics of fluorene (**a**), pyrene (**b**), and benzo(**a**)pyrene (**c**) without photocatalyst (*1*) and in the presence of photocatalysts TM1 (*2*), TM2 (*3*), and TM4 (*4*) at a 0–72 h contact time

By monitoring the *C*_t_/*C*_0_ ratio as a function of contact time, an initial high rate of degradation (0–6 h of contact time) was observed, whereas a longer contact time resulted in much slower or no further degradation (Fig. 5a–c). Specifically, during the initial 6 h, the degradation rates of fluorene, pyrene, and benzo(a)pyrene ranged from 16 to 67 µg/h, 13 to 74 µg/h, and 13 to 87 µg/h, respectively. The highest degradation rate was observed in the presence of TM1, and the lowest was observed in the presence of TM4. With further contact (6–72 h), the degradation rate decreased significantly (0.12–1.2 µg/h). In all cases, degradation followed first-order kinetics, the results of which are given in Table 4. Obtained results are in accordance with previously conducted studies by Luo et al. (2015b) and Chang Chien et al. (2011).

**Table 4:** Reaction kinetics parameters for the photodegradation of PAHs in the presence of TiO2/MgO nanocomposites

Material	PAH	*F*_fast_	*k*_fast_ (h^−1^)	*F*_slow_	*k*_slow_ (h^−1^)	*R*^2^
TM1	Fluorene	18.3	0.047	81.7	1.2·10^**−**3^	0.9973
	Pyrene	15.7	0.203	84.3	1.2·10^**−**3^	0.9693
	Benzo(α)pyrene	11.3	0.030	88.7	1.2·10^**−**3^	0.9656
TM2	Fluorene	36.8	0.040	68.3	2.9·10^**−**4^	0.9859
	Pyrene	26.4	0.039	73.6	8.8·10^**−**4^	0.9943
	Benzo(α)pyrene	30.4	0.029	63.3	4.5·10^**−**5^	0.9844
TM4	Fluorene	41.5	0.175	58.5	2.5·10^**−**4^	0.9473
	Pyrene	40.7	0.431	59.3	2.0·10^**−**4^	0.8391
	Benzo(α)pyrene	24.6	0.370	75.4	1.8·10^**−**4^	0.8261

The degradation rate constants (*k*_fast_, *k*_slow_) obtained through the kinetics study showed that *k*_slow_ was significantly lower than *k*_fast_, from 25 times for benzo(α)pyrene in the presence of TM1 to 2155 times for pyrene in the presence of TM4. The ratio of fast- to slow-degrading fractions of all PAHs (*F*_fast_/*F*_slow_) was < 1 (0.13‒0.71) in the presence of the studied photocatalysts, indicating that after 6 h, both the fast-degrading fraction and part of the slow-degrading fraction underwent a photocatalytic reaction.

## Conclusions

Using SHS, gerostructural mesoporous TiO_2_/MgO powders with a specific surface area and average pore diameter of 22.0–28.4 m^2^/g and 17–24 nm, respectively, were obtained. The highest degree of photocatalytic oxidation of PAHs in the presence of TiO_2_/MgO nanocomposites under the influence of daylight was achieved with a photocatalyst dose of 1 mg/L and at pH 7. The heterostructure system of oxide semiconductors TiO_2_/MgTi_2_O_5_/MgTiO_3_ showed a high degree of decomposition of fluorene, pyrene, and benzopyrene — 80, 68, and 53%, respectively. The high efficiency of the sample containing the MgTiO_3_, MgTi_2_O_5_, and TiO_2_ phases in the processes of PAH degradation is explained by the high content of titanium dioxide with a rutile structure and the separation of electron–hole pairs due to matched heterojunctions in the oxide semiconductor system. Degradation followed first-order kinetics, which included quick initial degradation along with slow or no degradation with a further increase in time.

## Data Availability

All data used to support the outcomes of the study are included in this article.

## References

[CR1] Aziz NAA, Palaniandya P, Moon WC, Khasawneh OFS, Aljuboury DADA (2021) Removal of fluoranthene and pyrene from rainwater using solar/TiO2 photocatalysis: optimization study. AIP Conf Proc 2332:P. 070001. 10.1063/5.0043753

[CR2] Bai H, Zhou J, Zhang H, Tang G (2017) Enhanced adsorbability and photocatalytic activity of TiO2-graphene composite for polycyclic aromatic hydrocarbons removal in aqueous phase. Colloids Surf. B Biointerfaces 150:68–77. 10.1016/j.colsurfb.2016.11.017 27886549 10.1016/j.colsurfb.2016.11.017

[CR3] Bakhsh EM, Khan ShB, Akhtar K, Danish EY, Fagieh TM, Qiu Ch, Sun Y, Romanovski V, Su X (2022) Simultaneous preparation of humic acid and mesoporous silica from municipal sludge and their adsorption properties for U(VI). Colloids Surf, A 647:129060. 10.1016/j.colsurfa.2022.129060

[CR4] Beljin J, Isakovski M. Kr., Zeremski T, Đukanović N, Apostolović T, Rončević S, Maletić S (2023) The efficiency of the hard wood origin biochar addition on the PAHs bioavailability and stability in sediment. J Hazard Mater Adv 10:100276. 10.1016/j.hazadv.2023.100276

[CR5] Bhagwat UO, Wu JJ, Asiri AM, Anandan S (2019) Synthesis of MgTiO_3_ nanoparticles for photocatalytic applications. Chemistry Select 4:788–796. 10.1002/slct.201803583

[CR6] Chang Chien SW, Chang CH, Chen SH, Wang MC, Madhava Rao M, Satya Veni S (2011) Effect of sunlight irradiation on photocatalytic degradation of pyrene in contaminated soils by micro-nano size TiO2. Sci Total Environ 409:4101–4108 21762957 10.1016/j.scitotenv.2011.06.050

[CR7] Charisio ND, Papageridis KN, Tzounis L, Sebastian V, Hinder SJ, Baker MA, AlKetbi M, Polychronopoulou K, Goula MA (2018) Ni supported on CaO-MgO-Al_2_O_3_ as a highly selective and stable catalyst for H_2_ production via the glycerol steam reforming reaction. Int J Hydrogen Energy 44(1):256–273. 10.1016/j.ijhydene.2018.02.165

[CR8] De Haart LGJ, de Vries AJ, Blasse G (1984) Photoelectrochemical properties of MgTiO_3_ and other titanates with the ilmenite structure. Mater Res Bull 19(7):817–824. 10.1016/0025-5408(84)90042-4

[CR9] Escandar GM, Muñoz de la Peña A (2021) Multiway calibration for the quantification of polycyclic aromatic hydrocarbons in samples of environmental impact. Microchem J 164:106016. 10.1016/j.microc.2021.106016

[CR10] Feng X, Feng J, Li W (2018) Insight into MgO promoter with low concentration for the carbon-deposition resistance of Ni-based catalysts in the CO2 reforming of CH4. Chinese Journal of Catalysis 39(1):88–98. 10.1016/S1872-2067(17)62928-0

[CR11] Ge L, Wang W, Peng Z, Tan F, Wang X, Chen J, Qiao X (2018) Facile fabrication of Fe@MgO magnetic nanocomposites for efficient removal of heavy metal ion and dye from water. Powder Technology 326:393–401. 10.1016/j.powtec.2017.12.003

[CR12] Glinskaya A, Petrov G, Vialikanava I, Romanovski V (2023) Crystal structure, magnetic and photocatalytic properties of solid solutions Bi2-xLaxFe4O9 (x = 0.05, 0.1). ChemistrySelect. 8(8):e202204285. 10.1002/slct.202204285

[CR13] Gupta SM, Tripathi M (2011) A review of TiO2 nanoparticles. Chinese Science Bulletin 56(16):1639–1657. 10.1007/s11434-011-4476-1

[CR14] Gurgenidze D, Romanovski V (2023) The pharmaceutical pollution of water resources using the example of the Kura River (Tbilisi, Georgia). Water 15(14):2574. 10.3390/w15142574

[CR15] Huang J, Li X, Wang X, Fang X, Wang H, Xu X (2019) New insights into CO2 methanation mechanisms on Ni/MgO catalysts by DFT calculations: elucidating Ni and MgO roles and support effects. Journal of CO2 Utilization. 33:55–63. 10.1016/j.jcou.2019.04.022

[CR16] Hyunwoong P, Yiseul P, Wooyul K, Wonyong C (2013) Surface modification of TiO2 photocatalyst for environmental applications. J Photochem Photobiol C: Photochem Rev 15:1–20. 10.1016/j.jphotochemrev.2012.10.001

[CR17] Khaled A, Richard C, Redin L, Niinipuu M, Janson S, Jaber F, Sleiman M (2018) Characterization and photodegradation of polybrominated diphenyl ethers in car seat fabrics from end-of-life vehicles. Environ Sci Technol 52(3):1216–1224. 10.1021/acs.est.7b04668 29261294 10.1021/acs.est.7b04668

[CR18] Kiani A, Nabiyouni Gh, Masoumi Sh, Ghanbari D (2019) A novel magnetic MgFe2O4–MgTiO3 perovskite nanocomposite: rapid photodegradation of toxic dyes under visible irradiation. Composites Part B. Eng 175:107080. 10.1016/j.compositesb.2019.107080

[CR19] Kulak A, Kokorin A (2023) Enhanced titania photocatalyst on magnesium oxide support doped with molybdenum. Catalysts. 13(3):454. 10.3390/catal13030454

[CR20] Li X, Huang Y, Zhang Q, Luan Ch, Vinokurov VA, Huang W (2019) Highly stable and anti-coking Ni/MoCeZr/MgAl2O4-MgO complex support. Energy Conversion and Management 179:166–177. 10.1016/j.enconman.2018.10.067

[CR21] Li H, Yu J, Gong Y, Lin N, Yang Q, Zhang X, Wang Y (2023) Perovskite catalysts with different dimensionalities for environmental and energy applications: a review. Separation and Purification Technology 307:122716. 10.1016/j.seppur.2022.122716

[CR22] Liu M, Wang Y, Chen L, Zhang Y, Lin Zh (2015) Mg(OH)2 supported nanoscale zero valent iron enhancing the removal of Pb(II) from aqueous solution. ACS Appl Mater Interfaces 7:7961–7969. 10.1021/am509184e 25826707 10.1021/am509184e

[CR23] Liu Z, Xu P, Song H, Xu J, Fu J, Gao B, Chu PK (2018) In situ formation of porous TiO_2_ nanotube array with MgTiO_3_ nanoparticles for enhanced photocatalytic activity. Surf Coat Int 365:222–226. 10.1016/j.surfcoat.2018.07.06

[CR24] Luo Z, Wei C, He N, Sun Z, Li H, Chen D (2015a) Correlation between the photocatalytic degradability of PAHs over Pt/TiO2-SiO2 in water and their quantitative molecular structure. J Nanomater 284834:284834. 10.1155/2015/284834

[CR25] Luo L, Lai X, Chen B, Lin L, Fang L, Tam NFY, Luan T (2015b) Chlorophyll catalyze the phototransformation of carcinogenic benzo[a]pyrene in water. Sci Rep 5:1–11. 10.1038/srep12776 10.1038/srep12776 PMC4523946 26239357

[CR26] Matsukevich I, Lipai Y, Romanovski V (2021) Cu/MgO and Ni/MgO composite nanoparticles for fast, high-efficiency adsorption of aqueous lead (II) and chromium (III) ions. J Mater Sci 56:5031–5040

[CR27] Matsukevich I, Kulak A, Palkhouskaya V, Romanovski V, Jo JH, Aniskevich Y, Mohamed SG (2022) Comparison of different methods for Li_2_MTi_3_O_8_ (M – Co, Cu, Zn) synthesis. J Chem Technol Biotechnol 97(4):1021–1026. 10.1002/jctb.6992

[CR28] Matsukevich I, Kulak A, Popkov V, Romanovski V, Fayed M, Mohamed S (2022) Lithium cobalt titanate with the spinel structure as an anode material for lithium-ion batteries. Inorg Mater 58(2):160–164. 10.1134/S0020168522010083

[CR29] Matsukevich I, Kulinich N, Kulbitskaya L, Kuznetsova T, Popkov V, Chebanenko M, Moskovskikh D, Kuskov K, Romanovski V (2023) Mesoporous nanocomposites based on CeO_2_ and MgO: preparation, structure, and photocatalytic activity. J Chem Technol Biotechnol 98:2497–2505. 10.1002/jctb.7476

[CR30] McQueen AD, Ballentine ML, May LR, Laber CH, Das A, Bortner MJ, Kennedy AJ (2021) Photocatalytic degradation of polycyclic aromatic hydrocarbons in water by 3D printed TiO2 composites. ACS EST Water 1–11 10.1021/acsestwater.1c00299

[CR31] Meng L, Ren Z, Zhou W, Qu Y, Wang G (2017) MgTiO_3_/MgTi_2_O5/TiO_2_ heterogeneous belt-junctions with high photocatalytic hydrogen production activity. Nano Res 10:295–304. 10.1007/s12274-016-1292-6

[CR32] Pawar TJ, Contreras LD, Olivares Romero JL et al (2023) Surface modification of titanium dioxide. J Mater Sci 58:6887–6930. 10.1007/s10853-023-08439-x

[CR33] Periakaruppan R, Romanovski V, Thirumalaisamy SK, Palanimuthu V, Sampath MP, Anilkumar A, Sivaraj DK, Ahamed NAN, Murugesan S, Chandrasekar D, Selvaraj KSV (2023) Innovations in modern nanotechnology for the sustainable production of agriculture. ChemEngineering 7(4):61. 10.3390/chemengineering7040061

[CR34] Pradhan G, Maurya S, Pradhan S, Sharma YCh (2023) An accelerated route for synthesis of glycerol carbonate using MgTiO_3_ perovskite as greener and cheaper catalyst. Mol Catal 545:113162(28). 10.1016/j.mcat.2023.113162

[CR35] Qu R, Li C, Liu J, Xiao R, Pan X, Zeng X, Wang Z, Wu J (2018) Hydroxyl radical based photocatalytic degradation of halogenated organic contaminants and paraffin on silica gel. Environ Sci Technol 52:7220–7229. 10.1021/acs.est.8b00499 29888912 10.1021/acs.est.8b00499

[CR36] Romanovski V, Matsukevich I, Romanovskaia E, Periakaruppan R (2023) Nano metal oxide as nanosensors in agriculture and environment. In Nanometal Oxides in Horticulture and Agronomy (pp. 321–352). Academic Press. ISBN: 9780323918091 10.1016/B978-0-323-91809-1.00016-0

[CR37] Romanovski V, Dubina A, Sehat AA, Su X, Moskovskikh D (2024a) Green and bio-waste-based materials for energy production, conversion, storage, and hybrid technologies. In: Materials for energy production, conversion, and Storage. CRC Press, Boca Raton, FL, USA, pp 118–135. https://www.taylorfrancis.com/chapters/edit/10.1201/9781003318859-8/green-bio-waste-based-materials-energyproduction-conversion-storage-hybrid-technologies-valentin-romanovski-alexandr-dubina-ali-akbari-sehat-xintai-sudmitry-moskovskikh

[CR38] Romanovski V, Pilipenko M, Dubina A, Likhavitski V, Volodko S, Moskovskikh D, Romanovskaia E (2024b) Optimizing dye wastewater purification: ultrasonic and flotation with ozonation synergy. Engineering Reports. 10.1002/eng2.13044

[CR39] Rotondo LN, Mora VC, Temporetti PF, Beamud SG, Pedrozo FL (2023) The use of an algal bioindicator in the assessment of different chemical remediation strategies for PAH-contaminated soils and sediments. J Environ Chem Eng 11(3):110098. 10.1016/j.jece.2023.110098

[CR40] Selvamani T, Anandan S, Asiri AM, Maruthamuthu P, Ashokkumar M (2021) Preparation of MgTi2O5 nanoparticles for sonophotocatalytic degradation of triphenylmethane dyes. Ultras Sonochem 75:105585. 10.1016/j.ultsonch.2021.105585 10.1016/j.ultsonch.2021.105585 PMC8182103 34087757

[CR41] Shaban YA (2019) Solar light-induced photodegradation of chrysene in seawater in the presence of carbon-modified n-TiO_2_ nanoparticles. Arab J Chem 12:652–663. 10.1016/j.arabjc.2018.01.007

[CR42] Sohara K, Yamauchi K, Sun X, Misawa K, Sekine Y (2021) Photocatalytic degradation of polycyclic aromatic hydrocarbons in fine particulate matter (PM2.5) collected on TiO2-supporting quartz fiber filters. Catalysts 11:1–12. 10.3390/catal11030400

[CR43] Soni H, Kumar N, Patel K, Kumar RN (2017) Investigation on the heterogeneous photocatalytic remediation of pyrene and phenanthrene in solutions using nanometer TiO_2_ under UV irradiation. Polycycl Aromat Compd 40:257–267. 10.1080/10406638.2017.1411956

[CR44] Thommes M, Kaneko K, Neimark AV, Olivier JP, Rodriguez-Reinoso F, Rouquerol J, Sing KS (2015) Physisorption of gases, with special reference to the evaluation of surface area and pore size distribution (IUPAC Technical Report). Pure Appl Chem 87(9–10):1051–1069

[CR45] Tu Z, Qi Y, Qu R, Tang X, Wang Z, Huo Z (2022) Photochemical transformation of hexachlorobenzene (HCB) in solid-water system: kinetics, mechanism and toxicity evaluation. Chemosphere. 295:P. 133907. 10.1016/j.chemosphere.2022.133907 10.1016/j.chemosphere.2022.133907 35151701

[CR46] Wang X, Cai J, Zhang Y, Li L, Jiang L, Wang Ch (2015) Heavy metal sorption properties of magnesium titanate mesoporous nanorods. J Mater Chem A 3:11796–11800. 10.1039/C5TA02034D

[CR47] Wang W, Zhang H, Wu L, Li J, Qian Y, Li Y (2016) Enhanced performance of dye-sensitized solar cells based on TiO2/MnTiO3/MgTiO3 composite photoanode. Journal of Alloys and Compounds 657:53–58. 10.1016/j.jallcom.2015.09.246

[CR48] Wang L, Yang G, Peng S, Wang J, Ji D, Yan W, Ramakrishna S (2017) Fabrication of MgTiO3 nanofibers by electrospinning and their photocatalytic water splitting activity. Int J Hydrogen Energy 42:25882–25890. 10.1016/j.ijhydene.2017.08.19

[CR49] Wang H, Wang Y, Liu Zh, Luo Sh, Romanovski V, Huang X, Czech B, Sun H, Li T (2022) Rational construction of micron-sized zero-valent iron/graphene composite for enhanced Cr(VI) removal from aqueous solution. J Environ Chem Eng 10(6):109004. 10.1016/j.jece.2022.109004

[CR50] Yang G, Wang L, Zhao Y, Peng S, Wang J, Ji D, Ramakrishna S (2018) One-dimensional Mg_x_Ti_y_O_x+2y_ nanostructures: general synthesis and enhanced photocatalytic performance. Appl Catal B 225:332–339. 10.1016/j.apcatb.2017.11.062

[CR51] Yang J, Yang H, Dong Y, Cui H, Sun H, Yin Sh (2023) Fabrication of Cu2O/MTiO3 (M = Ca, Sr and Ba) p-n heterojunction for highly enhanced photocatalytic hydrogen generation. J. All. and Comp. 930:P.167333. 10.1016/j.jallcom.2022.167333

[CR52] Zeng J, Xu R, El-Kady AA, Oranj BT, Ahmed R, Valentin R, Hu X, Wu W, Wang D, Mao J, Wu H, Gu X, Li P, Xu W, Zhang Z (2023) Nanomaterials enabled photoelectrocatalysis for removing pollutants in the environment and food. TrAC Trends in Analytical Chemistry 117187:117187. 10.1016/j.trac.2023.117187

[CR53] Zhang Y, Wong JWC, Liu P, Yuan M (2011) Heterogeneous photocatalytic degradation of phenanthrene in surfactant solution containing TiO2 particles. J. Hazard. Mater. 191:136–143. 10.1016/j.jhazmat.2011.04.059 21571431 10.1016/j.jhazmat.2011.04.059

[CR54] Zheng P, Hao R, Zhao J, Jia S, Cao B, Zhu Z (2014) Kinetic reconstruction of TiO2 surfaces as visible-light-active crystalline PAHs with highest photocatalytic performance. J Mater Chem A 2:4907–4911. 10.1039/C3TA15265K

[CR55] Zhou Z, Zhang L, Yan B, Wu J, Kong D, Romanovski V, Ivanets A, Li H, Chu S, Su X (2024) Removal of chromium from electroplating sludge by roasting-acid leaching and catalytic degradation of antibiotics by its residue. J Environ Chem Eng 12(1):111754. 10.1016/j.jece.2023.111754

